# Protein complex detection using interaction reliability assessment and weighted clustering coefficient

**DOI:** 10.1186/1471-2105-14-163

**Published:** 2013-05-20

**Authors:** Nazar Zaki, Dmitry Efimov, Jose Berengueres

**Affiliations:** 1 Intelligent Systems, College of Information Technology, UAEU, Al Ain, UAE; 2 Faculty of Mechanics and Mathematics, Moscow State Uni., Moscow, Russia

## Abstract

**Background:**

Predicting protein complexes from protein-protein interaction data is becoming a fundamental problem in computational biology. The identification and characterization of protein complexes implicated are crucial to the understanding of the molecular events under normal and abnormal physiological conditions. On the other hand, large datasets of experimentally detected protein-protein interactions were determined using High-throughput experimental techniques. However, experimental data is usually liable to contain a large number of spurious interactions. Therefore, it is essential to validate these interactions before exploiting them to predict protein complexes.

**Results:**

In this paper, we propose a novel graph mining algorithm (PEWCC) to identify such protein complexes. Firstly, the algorithm assesses the reliability of the interaction data, then predicts protein complexes based on the concept of weighted clustering coefficient. To demonstrate the effectiveness of the proposed method, the performance of PEWCC was compared to several methods. PEWCC was able to detect more matched complexes than any of the state-of-the-art methods with higher quality scores.

**Conclusions:**

The higher accuracy achieved by PEWCC in detecting protein complexes is a valid argument in favor of the proposed method. The datasets and programs are freely available at http://faculty.uaeu.ac.ae/nzaki/Research.htm.

## Background

Protein complexes are groups of associated polypeptide chains whose malfunctions play a vital role in disease development [1]. Complexes can perform various functions in the cell, including dynamic signaling, and can serve as cellular machines, rigid structures, and post-translational modification systems. Many disorders are consequences of changes in a single protein, and thus, in its set of associated partners and functionality. Therefore, mapping proteins and their interactions through the identification of protein complexes is a critical challenge in modern biology and can lead to significant applications for the diagnosis and treatment of diseases. Several outstanding computational approaches are developed to predict the structure of protein complexes from protein-protein interaction (PPI) networks. PPI is often modeled as the graph *G* = (*V*,*E*), where *V* is a set of nodes (proteins) and *E* is a set of edges (interactions) connecting pairs of nodes. A protein complex in this case is modeled as a dense subgraph of proteins, where the density is defined as the fraction of edges out of all possible vertex pairs. Two of the most frequently used algorithms for predicting protein complexes via the dense protein subgraph model are Markov clustering (MCL) [2] and repeated random walks (RRW) [3]. They both simulate random walks on the underlying PPI network. Another method is restricted neighborhood search clustering (RNSC), [4,5] which uses principles of local search algorithms such as restricted neighborhood search, tabu search, and diversification schemes to ensure good performance and speed. Leung et al. [6] developed an algorithm called Core based on the core-attachment idea, and Zaki et al. [7] recently proposed a novel method for detecting protein complexes in PPI based on a protein ranking algorithm (ProRank). ProRank quantifies the importance of each protein based on the interaction structure and evolutionary relationships between proteins in the network. Methods based on protein clustering with overlapping neighborhood expansion, such as CFinder [8], which is one of the oldest overlapping clustering methods and the recently published method known as ClusterONE [9] have also been introduced. The ClusterONE method initiates from a single seed vertex before a greedy growth procedure begins to add or remove vertices in order to find groups with high cohesiveness. The cohesiveness is defined as follows: Let *w*^*in*^(*V*) and *w*^*bound*^(*V*) denote the total weight of edges contained entirely by a group of proteins *V*, and the total weight of edges that connect the group with the rest of the network, respectively. Following [9], the cohesiveness of *V* is then given by $f(V)=\frac{w^{\text{in}}(V)}{w^{\text{in}}(V)+w^{\text{bound}}(V)+p|V|}$, where *p*|*V*| is a penalty term whose purpose is to model the uncertainty in the data by assuming the existence of yet undiscovered interactions in the protein interaction network. ClusterONE-derived complexes from various yeast datasets and managed to show better agreement with reference complexes drawn from the Munich Information Center for Protein Sequence (MIPS) catalog and the Saccharomyces Genome Database (SGD) than the results of several other popular methods. However, one weakness in the process is that it is dependent on the quality of the PPI data mainly produced by high-throughput experiments. Such experiments are believed to be noisy and fragmented due to the limitations of the corresponding experimental techniques and the dynamic nature of protein interaction maps, which may have a negative impact on the performance of complex recognition algorithms [10]. For example, it is thought that the false positive rate of Y2H screens could be as high as 64%, and the false negative rate can vary from 43% to 71% [11]. Sprinzak et al. [12] showed that the reliability of high-throughput yeast two-hybrid assays is around 50%, and that the size of the yeast interactome is estimated to be 10,000 to 16,000 interactions. Xiaoli Li et al. [13] have also shown that improvement in protein complex detection could be achieved if the quality of the underlying PPI data is considered adequately to minimize the undesirable effects from the irrelevant and noisy sources. To solve this problem, several methods, such as the molecular complex detection (MCODE) algorithm [14], was proposed to assess the reliability of high-throughput protein interaction data. The MCODE algorithm depends on the vertex weighting phase in which a score is assigned to each vertex (measuring the cliquishness of the neighborhood of the vertex). The vertex weight percentage controls how much difference is allowed between the scores of the vertices within the same complex and those outside the complex. By proposing weighting schemes based on the number of common neighbors, other authors were able to improve several clustering algorithms such as CDdistance [15] and FSWeight [16]. To this end, Liu et al. [10] have recently developed an algorithm, referred to as Clustering, which is based on Maximal Cliques (CMC) for discovering protein complexes in weighted PPI networks. They used an iterative scoring method called AdjstCD to assign weights to protein pairs. The AdjstCD weight in this method indicates the reliability of the interaction between protein pairs. The AdjstCD iterative algorithm [15,17,18] is mainly based on the number of common protein-pair neighbors in the PPI network. The CD-distance [17] between two neighbor proteins *u* and *v* is defined as:

(1) $$\text{CD}(u,v)=1-\frac{2|N_{u}\cap N_{v}|}{\left|N_{u}|+|N_{v}\right|}$$

where *N*_*u*_ and *N*_*v*_ are the numbers of neighbors of proteins *u* and *v*, respectively. Equation (1) was further modified by Chua et al. [18] to decrease the CD-distance for proteins with insufficient number of interactions:

(2) $$\text{AdjstCD}(u,v)=\frac{2|N_{u}\cap N_{v}|}{\text{max}(|N_{u}|,N_{\text{avg}})+\text{max}(|N_{v}|,N_{\text{avg}})}$$

where $N_{\text{avg}}=\frac{\underset{x\in V}{\sum }|N_{x}|}{N}$ is the average number of neighbors in the network and *N* is the total number of nodes in the network.

**Figure 1:**
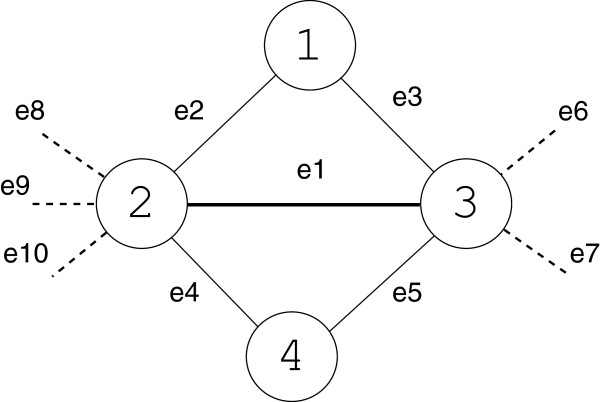
**Reliability weight of the edge *e*1 using AdjstCD depends on the outgoing edges *e*6,*e*7, …,*e*10.** However, in a case of noisy network there is a possibility that many of these outgoing edges may not be reliable. Therefore, the reliability of the edge *e*1 should not be influenced by all of the outgoing edges.

Equations (1) and (2), show how many 3-cliques can be generated from the interactions between proteins *u* and *v*, but do not take into account groups of the 3-cliques based on other outgoing interactions from proteins *u* and *v*. To solve this problem, Chua et al. [18] suggested an iterative method which considers all 3-cliques from all neighbor proteins *u* and *v*:

(3) $$\begin{array}{l} w^{k}\;(u,v)\\ \;=\frac{\underset{x\in N_{u}\cap N_{v}}{\sum }(w^{k-1}(x,u)+w^{k-1}(x,v))}{\text{max}(\underset{x\in N_{u}}{\sum }w^{k-1}(x,u),w_{\text{avg}}^{k-1})+\text{max}(\underset{x\in N_{v}}{\sum }w^{k-1}(x,v),w_{\text{avg}}^{k-1})} \end{array}$$

where *w*^0^(*x*,*u*) = 1, if *x* and *u* interact, *w*^0^(*x*,*u*) = 0, otherwise; $w_{\text{avg}}^{k-1}=\frac{\underset{x\in V}{\sum }\underset{y\in N_{x}}{\sum }w^{k-1}(x,y)}{n}$ is the average number of weights at (*k*−1)^*th*^ step; *w*^1^(*x*,*u*) = *AdjstCD*(*x*,*u*) and eventually *w*^*k*^(*u*,*v*) will determine the reliability of interaction between proteins *u* and *v*. It was shown that the iterative scoring method can significantly improve the performance of CMC and some other well known protein complex detection methods such as MCL [2],CFinder [8] and MCODE [14]. However, CMC works accurately on reasonably clean protein interaction data (few missing interactions). It is quite difficult to identify unreliable edges or to find maximal cliques when the data is noisy. This weakness is demonstrated by Figure 1. The reliability weight of the edge *e*1 using AdjstCD depends on the outgoing edges *e*6,*e*7,…,*e*10. In a case of noisy network there is a possibility that many of the outgoing edges such as *e*6,*e*7,…,*e*10 may not be reliable. Moreover, the reliability of the edge *e*1 should not be influenced by all of the outgoing edges.

In this paper, we propose a simple yet effective method for protein complex identification. We are aware of the fact that, in addition to improving graph mining techniques, it is necessary to obtain high quality benchmarks by assessing protein interaction reliability. Therefore, we propose a novel method for assessing the reliability of interaction data and detecting protein complexes. Unlike CMC, this method finds near-maximum cliques (maximal cliques without unreliable interactions). We employ the concept of weighted clustering coefficients as a measure to define which subgraph is the closest to the maximal clique. The clustering coefficient of a vertex in this case is the density of its neighborhood [19].

## Methods

Computational approaches for detecting protein complexes from PPI data are useful complements to the limitation of the experimental methods such as Tandem Affinity Purification (TAP) [20]. Beside the improvement in graph mining techniques, the success of accurate detection of a protein complex depends on the availability of high-quality benchmarks. The bottleneck of different computational methods remains to be the noise associated with the protein interaction data. Therefore, a rigorous assessment of protein interactions reliability is essential. In this section, we introduce a novel method PEWCC which has two main steps: first, assess the reliability of the protein interaction data using the PE-measure. Second, detect protein complexes using weighted clustering coefficient [19,21] (WCC). In the subsequent sections, we describe these two steps in details.

### Assessing the reliability of protein interactions

In this section we introduce the PE-measure, a new measure for protein pairs interaction reliability. PE-measure enables us to reduce the level of noise associated with PPI networks and it is defined as follows:

Given a PPI network with *N* proteins, we represent the PPI network by an undirected graph *G* = (*V*,*E*), where the vertex set *V* represents the proteins, and the edge set *E* represents the set of interactions between pairs of proteins. The elements (*p*_0_)_*ij*_ of the initial (*N *×* N*) reliability matrix *P*_0_ are equal to 0.5 (given that *i* interacts with *j*). We then calculate the elements (*p*_*k*_)_*ij*_ of the matrix *P*_*k*_ in *k* iterations as:

(4) $${\left(p_{k}\right)}_{\text{ij}}=1-\underset{v_{l}}{\prod }(1-{\left(p_{k-1}\right)}_{\text{il}}\cdot {\left(p_{k-1}\right)}_{\text{jl}})$$

where we take the product by all *v*_*l*_ : (*v*_*l*_,*v*_*i*_) ∈ *E*,(*v*_*l*_,*v*_*j*_) ∈ *E*.

**Figure 2:**
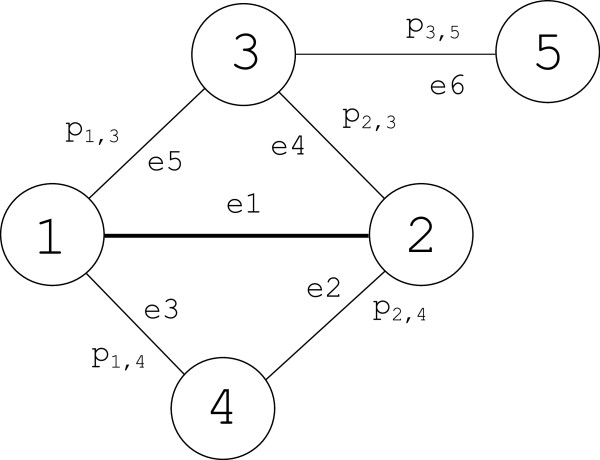
**A simple hypothetical network of 5 proteins and 6 interactions to illustrate how the weight of the edge *e***_**1**_** is determined.**

To illustrate the weighting scheme, consider a hypothetical network as shown in Figure 2.

Suppose we would like to determine the weight of the edge *e*_1_ (between protein 1 and protein 2). According to Equation (4), the probabilities that protein 3 and protein 4 do not “support” the edge *e*_1_ are (1−*p*_1,3_·*p*_2,3_) and (1−*p*_1,4_·*p*_2,4_), respectively. Thus, the probability that protein 3 and 4 do not “support” the edge *e*_1_ is (1−*p*_1,3_·*p*_2,3_)·(1−*p*_1,4_·*p*_2,4_). Therefore, the probability that protein 1 and protein 2 interact (and supported by protein 3 and protein 4) is the complementary probability 1−[(1−*p*_1,3_·*p*_2,3_)·(1−*p*_1,4_·*p*_2,4_)].

We start with the initial probability matrix *P*_0_ (where *p*_1,3_, *p*_2,3_, *p*_2,4_, *p*_1,4_ and *p*_3,5_ are all equal to 0.5). In the first iteration (*k* = 1) the PE-measure of the edge *e*_1_ is $1-[(1-(p_{1,3}.p_{2,3})\cdot (1-(p_{1,4}\cdot p_{2.4})]=\frac{7}{16}$. Similarly, the PE- measures of edges *e*_2_, *e*_3_, *e*_4_ and *e*_5_ are all equal to $\frac{1}{4}$ while the measure of edge *e*_6_ is equal to 0. All of the PE-measures are updated before the second iteration (*k *= 2) starts.

For each protein in the PPI network, we calculate the average PE-measures (*w*_*avg*_)_*i*_ of all outgoing edges as follows:

(5) $${\left(w_{\text{avg}}\right)}_{i}=\frac{\underset{v_{l}}{\sum }p_{\text{il}}}{N_{i}},$$

where *v*_*l*_ : (*v*_*l*_,*v*_*i*_) ∈ *E*, *N*_*i*_ is the number of the neighbors of *v*_*i*_ and *i *= 1,…,*N*. If the PE-measure *p*_*il*_ is less than the average (*w*_*avg*_)_*i*_ then the edge between proteins *i* and *l* is considered unreliable and therefore, it should be removed from the network.

Applying Equation (4) on the hypothetical network shown in Figure 2, we could see that the edge *e*_6_ yields a lower weight which is equal to 0 and therefore, it could be a noise and should be removed from the network.

### Detecting protein complex using weighted clustering coefficient

**Figure 3:**
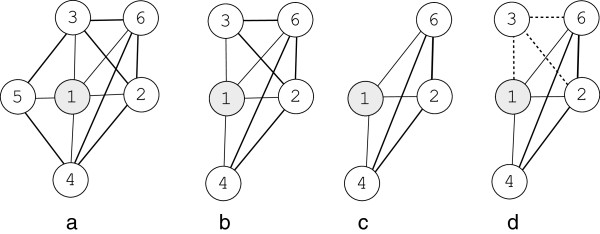
Illustration of how a protein complex is detected: (a) A simple hypothetical network of 6 proteins and 12 interactions, (b) based on the sequence of the degree, node 5 has only 2 outgoing connections and therefore, it is removed from the protein network, (c) based on the sequence of the degree, node 3 is removed and therefore, the subgraph which contains the central protein 1 and three nodes (2,4 and 6) remains as a valid core protein complex, (d) protein which interacts with more than 50% such as protein 3 rejoins the protein network and the final complex is predicted.

For each protein *v*_*i*_ in the PPI network, we first create the neighborhood graph, calculate the weighted clustering coefficient and then calculate the degree of each node in the neighborhood graph; the “degree” of a node being the number of its neighbors. The weighted clustering coefficient *c*_*i*_ in this case is calculated according to the following formula:

(6) $$c_{i}=\frac{2\cdot N_{3\text{cliques}}}{N_{i}^{2}\cdot (N_{i}-1)}$$

where *N*_3*cliques*_ is the number of 3-cliques in the neighborhood graph. Once the degree is calculated, we sort the sequence of proteins in the neighborhood graph accordingly from minimum to maximum. The protein *v*_*j*_ with the lowest degree and its corresponding interactions are removed from the neighborhood graph and *c*_*i*_ is recalculated. This process stops when the neighborhood graph contains only 3 proteins and the sequence of proteins with the highest *c*_*i*_ is returned as a valid core protein complex. This concept is illustrated in Figure 3.

In Figure 3 (a), if *i *= 1 then *N*_1_ in this case is equal to 5 (the central protein 1 is not considered), *N*_3*cliques*_ = 7 and therefore, according to Equation 6, $c_{1}=\frac{2\times 7}{5^{2}\times (5-1)}=0.14$. Based on the sequence of the degree, node 5 has only 2 outgoing connections and therefore, it should be removed from the subgraph. In Figure 3 (b), the subgraph is now reduced to 4 nodes, *N*_3*cliques*_ = 5 and therefore, *c*_1_ = 0.21. Based on the sequence of the degree there exists a tie and therefore either nodes 3 or 4 should be randomly removed. If the node 3 is removed as shown in Figure 3 (c) then we end up with a subgraph with only 3 nodes. The *c*_1_ in this case is equal to 0.33 and therefore, the subgraph which contains the central protein 1 and three nodes (2, 4 and 6) is a valid core protein complex. Once the core protein complex is identified, we examine the main subgraph once again and re-join any protein which interacts with more than *t%* of the proteins in the core protein complex. In the case of *t* = 50, protein 3 will join the subgraph and the final complex predicted is shown in Figure 3 (d).

### Assessing the quality of predicted complexes

To evaluate the accuracy of the proposed method, we used the Jaccard index which defined as follows:

(7) $$\text{MatchScore}(K,R)=\frac{\left|V_{K}\cap V_{R}\right|}{\left|V_{K}\cup V_{R}\right|}$$

where *K* is a cluster and *R* is a reference complex. *V*_*K*_ and *V*_*R*_ are the set of proteins in *K* and *R*, respectively. The complex *K* is defined to match the complex *R* if *MatchScore*(*K*,*R*) ≥ *α* where *α* = {0.25,0.5,0.6,0.7,0.8 or 0.9} (because different methods were evaluated with different values of *α*).

To estimate the cumulative quality of the prediction, assume a set of reference complexes *R *= {*R*_1_,*R*_2_,…,*R*_*n*_} and a set of predicted complexes *P* = {*P*_1_,*P*_2_,…,*P*_*m*_} the recall (*Rec*) and precision (*Prec*) at the complex level are then computed as follow:

(8) $$\text{Rec}=\frac{\left|\{R_{i}|R_{i}\in \mathrm{R\Lambda}\exists P,K_{j}\text{matches}R_{i}\}\right|}{\left|R\right|}$$

and

(9) $$\text{Prec}=\frac{\left|\{K_{j}|K_{j}\in P\Lambda \exists R_{i}\in R,R_{i}\text{matches}K_{j}\}\right|}{\left|P\right|}$$

Following Brohee and van Helden [22] and Nepusz et al. [9], we used the geometric mean of two other measures, namely the clustering-wise sensitivity (*Sn*) and the clustering-wise positive predictive value (*PPV*). Both *Sn* and *PPV* are based on the confusion matrix *T *= [*t*_*ij*_] of the complexes. Given *n* reference and *m* predicted complexes, let *t*_*ij*_ denote the number of proteins that are found both in reference complex *i* and predicted complex *j*, and let *N*_*i*_ denote the number of proteins in reference complex *i*. *Sn* and *PPV* are then defined as follows:

(10) $$\text{Sn}=\frac{\overset{n}{\underset{i=1}{\sum }}\text{ma}x_{j-1}^{m}t_{\text{ij}}}{\overset{n}{\underset{i=1}{\sum }}n_{i}}$$

and

(11) $$\text{PPV}=\frac{\overset{m}{\underset{j=1}{\sum }}\text{ma}x_{i-1}^{n}t_{\text{ij}}}{\overset{m}{\underset{j=1}{\sum }}\overset{n}{\underset{i=1}{\sum }}t_{\text{ij}}}$$

Since *Sn* can be inflated by putting every protein in the same cluster, while *PPV* can be maximized by putting every protein in its own cluster, the accuracy (*Acc*), which is simply the geometric mean of the clustering-wise sensitivity and the positive predictive value was defined as follows:

(12) $$\text{Acc}=\sqrt{\text{Sn}\times \text{PPV}}$$

Following Nepusz et al. [9], we also evaluated our method using the maximum matching ratio (MMR). The MMR measure is based on a maximal one-to-one mapping between predicted and reference complexes. The motivation for Nepusz et al. [9] to use the MMR is the fact that the PPV tends to be lower if there are substantial overlaps between the predicted complexes, which could limit the prediction accuracy when using overlapping clustering algorithms. The algorithm used to calculate the MMR is available in the supplementary material (Additional file 1).

The experimental works were conducted on a PC with Intel(R) Core(TM)2, CPU 6400 @ 2.13GHz and 3 GB of RAM.

## Results and discussion

In this section, we first describe the datasets and evaluate the current methods for protein complex detection, and then study the performance of PEWCC and the impact of the PE-measure. The effectiveness of our method is evaluated using two different PPI datasets. The first is a combined PPI dataset (PPI-D1) developed by Liu et al. [10] and it contains yeast protein interactions generated by six different experiments, including interactions characterized by the mass spectrometry technique [23-26], and interactions produced using two-hybrid techniques [27,28]. The second dataset (PPI-D2) is an entire set of physical protein-interaction in yeast from BioGRID [29]. The properties of the PPI-D1 and PPI-D2 datasets used in the experiments are shown in Table 1.

**Table 1:** Properties of the two PPI datasets used in the experimental work

**Dataset**	**Proteins**	**Interactions**	**Network density**	**Clustering coefficient**	**Av. no. of neighbors**	**Isolated proteins**
PPI-D1	3,869	19,165	0.002	0.157	8.957	8
PPI-D2	5,640	59,748	0.004	0.246	21.187	0

Three reference sets of protein complexes are used in these experiments. The first set of complexes (Cmplx-D1) comprises of 162 hand-curated complexes from MIPS [30]. The second dataset (Cmplx-D2) which contains 63 complexes is generated by Aloy et al. [31]. The third reference set (Cmplx-D3) of 203 complexes was developed by Nepusz [9] and it consists of the most recent version of the MIPS catalog of protein complexes. Both datasets Cmplx-D1 and Cmplx-D2 were used by Liu et al. [10] to evaluate the performance of the CMC method. Complexes with sizes greater or equal to 4 proteins were considered.

**Figure 4:**
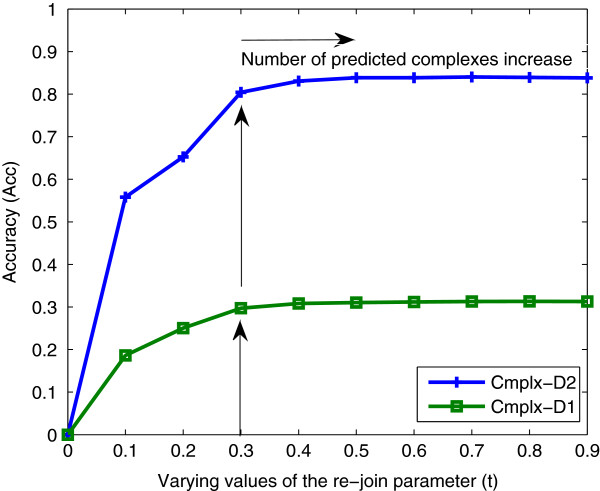
**Measuring the effect of varying the values of the re-join parameter (*t*) in terms of *Acc*.** For *t* > 0.3 we will still obtain similar accuracy however, increasing the value of *t* will increase the number of complexes detected and therefore, *t* is considered equal to 0.3.

In the first experimental work, we attempted to find the optimal value of the re-join parameter *t* which will lead to the best performance of the proposed method. In Figure 4, we show the effect of varying parameter *t* and the corresponding complex detection accuracy measured in terms of *Acc*. Based on PPI-D1 and the reference datasets Cmplx-D1 and Cmplx-D2, the results show that the best performance of the proposed method is achieved when *t *≥ 0.3. For *t *> 0.3 we will still obtain similar accuracy. However, increasing the value of *t* will increase the number of complexes detected which will decrease the *Prec*. Therefore, in all the following experimental works *t* was considered to be equal to 0.3. The parameter *k* (number of iterations) was set to 2 in all the experiments since no significant performance improvement was achieved when *k *> 2.

In Table 2 we compare the performance of the PEWCC method to CMC [10], ClusterONE [9], MCL [2], CFinder [8] and MCODE [14] based on PPI-D1 and different reference datasets Cmplx-D1 and Cmplx-D2. In this case more than one quality score were used to assess the performance of each algorithm: *Prec*, *Rec* and *F*1 (where $F1=2\cdot \frac{\text{Prec}\cdot \text{Rec}}{\text{Prec}+\text{Rec}}$). The fraction of matched complexes with a given overlap score threshold *Acc*(*K*,*P*) was set to be ≥ 0.5. For each algorithm, final results were obtained after optimizing the algorithm parameters to yield the best possible results. For the CMC, the algorithm is primarily affected by the overlap threshold and the merge threshold. The overlap threshold determines the highly overlapping score between two clusters and the merge threshold which determines what to do with two highly overlapping clusters (merged or removed). The overlap threshold and the merge threshold shown good performance when both were set to 0.5 and 0.25, respectively. The iterative scoring parameter *k* was set to 2. For ClusterONE, we used the default parameters density threshold set to 0.5. The merging threshold was set to 0.8 and the penalty value of each node was 2. The MCL has a single parameter called inflation, which tunes the granularity of the clustering and it was set to 1.8. For MCODE, the depth was set to 100, node score percentage to 0, and percentage for complex fluffing to 0.2 (as suggested by [22]). For CFinder, we set *k*-clique size to 4. The rest of the parameters were set to their default values. The summary of the parameters setup for all the methods is available in the supplementary materials (Additional file 2).

**Table 2:** **Performance comparison of PEWCC, CMC, ClusterONE, MCL, CFinder, and MCODE, with *A c c *( *K *, *P *) ≥ 0 *. *5**

	**Cmplx-D1**				**Cmplx-D2**			
**Method**	**Matched Cmplx**	***Prec***	***Rec***	**F1**	**Matched Cmplx**	***Prec***	***Rec***	**F1**
PEWCC	58	0.435	0.469	0.451	61	0.468	0.910	0.618
CMC	56	0.297	0.346	0.320	57	0.385	0.889	0.537
ClusterONE	52	0.204	0.387	0.267	48	0.231	0.872	0.365
MCL	51	0.353	0.315	0.333	52	0.448	0.825	0.581
MCODE	39	0.330	0.241	0.279	34	0.386	0.540	0.450
CFilter	46	0.379	0.284	0.325	43	0.463	0.683	0.552

As shown in Table 2, the proposed method was able to detect more matched complexes than any of the state-of-the-art methods with higher *F*1 value.

**Figure 5:**
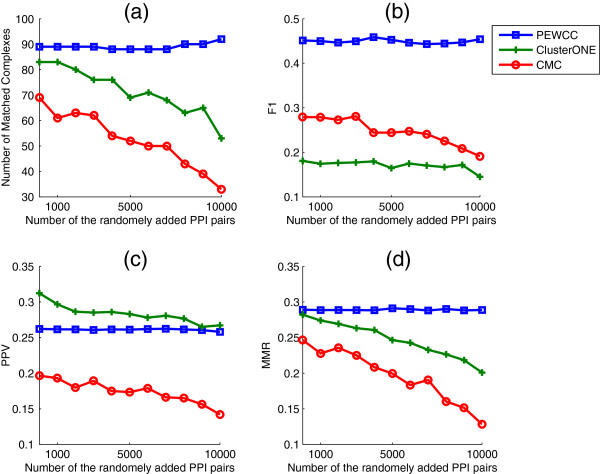
**Comparing PEWCC, ClusterONE and CMC in the presence of additional sets of random PPI pairs in terms of the number of matched complexes detected, F1, PPV and *MMR* scores.**

To analyze the performance of PEWCC, ClusterONE and CMC in a noisy interaction dataset, we added different random sets of interaction pairs to Cmplx-D1 (1000 PPI pairs at a time). In Figure 5 (a), we show the number of matched complexes detected using PEWCC, ClusterONE and CMC in the presence of different sets of random interaction pairs. In Figures 5 (b), (c) and (d) we compare the performances of the three mentioned methods in terms of the number of matched complexes *F*1, *PPV* and *MMR* scores respectively. The solid performance of PEWCC is quite obvious in the existence of additional sets of random interaction pairs (noise). The performances of ClusterONE and CMC deteriorated when the noise increases. In Figure 5 (c), ClusterONE showed better *PPV* score than PEWCC however, the latest showed consistent performance.

Furthermore, the impacts of the PE-measure and the AdjstCD measure on improving the detection of matched complexes were assisted using the datasets PPI-D1 and Cmplx-D1. In Table 3 we show the performance of CMC and ClusterONE with and without filtering method such as AdjstCD and PE measures. In this case the performances of CMC and ClusterONE in conjunction with the PE measures were significantly improved.

**Table 3:** **The performance of CMC and ClusterONE with and without filtering method such as AdjstCD and PE measures with *A c c *( *K *, *P *) ≥ 0 *. *5**

**Method**	**Clusters predicted**	**Matched Cmplx**	**Perc. of successful Cmplx**	***Rec***	***Prec***	***PPV***	***F*1**
CMC	133	45	28	0.217	0.263	0.172	0.238
ClusterONE	498	77	47.5	0.372	0.118	0.301	0.180
AdjstCD+CMC	127	75	46.3	0.362	0.455	0.277	0.404
AdjstCD+ClusterONE	139	78	48.2	0.377	0.393	0.294	0.385
PE+CMC	112	77	47.5	0.372	0.446	0.313	0.406
PE+ClusterONE	110	81	50	0.391	0.464	0.318	0.424
PE+WCC	128	89	54.9	0.435	0.469	0.262	0.451

For generalization purposes PEWCC was further compared to several state-of-the-art methods based on the protein interaction dataset PPI-D2 and the reference dataset Cmplx-D3. PPI-D2 and Cmplx-D3 were recently published and used to evaluate the performance of ClusterONE [9] in detecting protein complexes. In this case more than one quality score were used to assess the performance of each algorithm: following [9] the fraction of matched complexes with a given overlap score threshold *Acc*(*K*,*P*) ≥ 0.25 and the geometric accuracy. The performance of methods such as (RNSC) [4,5] and (RRW) [3] were included in the comparison. Please note that RNSC algorithm does not take into consideration the weights of the PPI graph edges. The summary of the parameters setup for all the methods used in the comparison is available in the supplementary materials (Additional file 2).

As shown in Table 4, the PEWCC method was able to detect more matched complexes (122 matching complexes) than any of the state-of-the-art methods with higher quality scores. It takes approximately 22 and 48 seconds for PEWCC to detect complexes from PPI-D1 and PPI-D2, respectively.

**Table 4:** **Compare PE-WCC to ClusterONE, RNSC, RRW, CMC, MCL and MCODE, where *A c c *( *K *, *P *) ≥ 0 *. *25**

**Method**	**Clusters predicted**	**Matched Cmplx**	**Perc. of successful Cmplx**	***Sn***	***PPV***	***Acc***	**MMR**
PEWCC	468	122	60.1	0.551	0.430	0.491	0.348
ClusterONE	473	88	43.3	0.454	0.427	0.440	0.195
RNSC	209	79	38.9	0.399	0.441	0.419	0.192
RRW	253	75	36.9	0.276	0.429	0.344	0.178
CMC	73	53	26.1	0.323	0.404	0.487	0.176
MCL	338	37	18.2	0.346	0.350	0.348	0.083
MCODE	85	21	10.3	0.285	0.284	0.285	0.048

## Conclusion

In this paper, we have provided a novel method (PEWCC) for detecting protein complexes from a PPI network of yeast. We have shown that our approach, which first assesses the quality of the interaction data and then detect the protein complex based on the concept of weighted clustering coefficient, is more accurate than most of the well known methods.

The noise associated with the PPI network and the focus on dense subgraphs have restricted researchers from creating an effective algorithm that is capable of identifying small complexes and PEWCC is no exception. In fact, we cannot recall any method that can effectively detect complexes (≤ 3 proteins) using only the topology of the PPI network. We understand that PEWCC stops when the neighborhood graph contains only 3 proteins which restricts it from identifying small complexes (≤ 3 proteins). It was possible for us to discover the clustering coefficient was *c*_*i*_ = 1 for dense graphs of size 3 (with 3 nodes and 3 edges) and *c*_*i*_ = 0 for other subgraphs of size 3 (with 3 nodes and 2 edges). We are currently conducting a systematic research of nested complexes (the case where one complex is a sub-complex of a bigger one) in order to identify strategies that could be useful in improving the capability of PEWCC in identifying small complexes.

The performance of PEWCC can also be tested when the edges were randomly removed from the original graph. However, we strongly believe that the main issue concerning PPI data is the noise associated with false interactions (edges). There are many interactions that are not reliable and by removing them, the prediction accuracy was improved by using PE measure and AdjstCD. Moreover, if we remove edges uniformly over the PPI network, then the PEWCC algorithm will still work, because it calculates relative density (one subgraph with respect to another). It means that if we have two subgraphs *G*_1_ and *G*_2_ and the density of *G*_1_ is less than the density of *G*_2_, then following the random deletion of some edges from *G*_1_ and *G*_2_, the probability that the density of *G*_1_ will be less than the density of *G*_2_, will still be very high.

In the future, we would like to compare the performance of PE to the recently published novel weighting schemes for noise reduction in PPI network by graphs by Kritikos et al. [32]. In this research work, only the topological properties of PPI graphs were taken into consideration while it has been proved that integrating additional biological knowledge helps the weighting schemes to generate more reliable PPI graphs. Therefore, an interesting open challenge is to study the incorporation of additional biological knowledge of protein complexes. To this end, a probabilistic calculation of the affinity score between two proteins [33] could further improve the performance of the proposed method.

Furthermore, the idea of decomposing the PPI network into overlapping clusters will be explored as it shows great potential in recent works [9,34-36].

## Competing interests

The authors declare that they have no competing interests.

## Authors’ contributions

NZ and DF designed the method and conceived the study. JB implemented the method. NZ performed the experiments and wrote the paper. All authors read and approved the final manuscript.

## Supplementary Material

Additional file 1 The algorithm to calculate the MMR. Click here for file

Additional file 2 The summary of the parameters setup. Click here for file
