# Supplementary material for: Protein complex detection using interaction reliability assessment and weighted clustering coefficient
Source: BMC Bioinformatics. 2013 May 20;14:163. doi: 10.1186/1471-2105-14-163 (PMC3680028; doi:10.1186/1471-2105-14-163)
Supplement: Additional file 1 — The algorithm to calculate the MMR. [file 1471-2105-14-163-S1.pdf]

Protein Complex Detection using Interaction Reliability Assessment and Weighted Clustering Coefficient

The algorithm to calculate the MMR is shown in Algorithm 1.

Algorithm 1 Algorithm for calculating MMR

```
Maximum_Matching_Ratio(reference, predicted, score_threshold)
n = size(reference)
for i = 1 to size(reference) do
    cmplx1 = reference[i]
    for j = 1 to size(predicted) do
        cmplx2 = predicted[j]
        matching_score = size(cmplx_intersection)^2 / size(cmplx1)=size(cmplx2)
        if matching_score ≤ score_threshold then
            continue
        end if
        scores[i, j + n] = matching_score
    end for
end for
for i = 1 to size(scores) do
    for j = 1 to size(scores) do
        add (i, j, scores[i, j]) to input          ▷ syntax may differ based on the declaration of "input"
    end for
end for
mates = Max_Weight_Matching(input)                 ▷ Max_Weight_Matching returns a list, here mates, where
                                                   ▷ mates[i] = j if i is matched to j and mates[i] = −1 otherwise:
for k = 1 to size(mates) do
    if k < mates[k] then
        score = score + scores[k, mates[k]]
    end if
end for
return score / n
```
