# Supplementary material for: Protein complex detection using interaction reliability assessment and weighted clustering coefficient
Source: BMC Bioinformatics. 2013 May 20;14:163. doi: 10.1186/1471-2105-14-163 (PMC3680028; doi:10.1186/1471-2105-14-163)
Supplement: Additional file 2 — The summary of the parameters setup. [file 1471-2105-14-163-S2.pdf]

Protein Complex Detection using Interaction Reliability Assessment and Weighted Clustering Coefficient

The summary of the parameters setup for all the methods used in the camoparison are shown in Table 1 and Table 2, respectively.

Table 1: The summary of the parameters setup for the methods used in the study.

Method	Parameter	Parameter Value
CMC	Overlap threshold	0.5
	Merging threshold	0.25
ClusterOne	Density threshold	0.5
	merging threshold	0.8
	penalty value	2
MCL	Inflation	1.8
MCode	Depth limit	0
	complex fluffing	0.2
CFinder	k-clique template size	4

Table 2: The summary of the parameters setup for the methods used in the study.

Method	Parameter	Parameter Value
ClusterOne	Density threshold	0.6
	merging threshold	0.8
	penalty value	2
RNSC	Shuffling diversification length	9
	Diversification frequency	20
	Number of experiments	3
	Naive stopping tolerance	20
	Scaled stopping tolerance	5
	Tabu length	10
	Tabu tolerance	1
RRW	Restart probability	0.9
	Overlap threshold	0.2
	Early cutoff	0.6
CMC	Overlap threshold	N/A
	Merge threshold	N/A
MCL	Inflation	3.3
MCode	Depth limit	3
	complex fluffing	no
